# Anabolic Strategies to Augment Bone Fracture Healing

**DOI:** 10.1007/s11914-018-0440-1

**Published:** 2018-05-03

**Authors:** Scott J. Roberts, Hua Zhu Ke

**Affiliations:** Bone Therapeutic Area, UCB Pharma, 208 Bath Road, Slough, Berkshire SL1 3WE UK

**Keywords:** Bone fracture, Non-union, Anabolic pathways, Endochondral ossification

## Abstract

**Purpose of Review:**

The development of therapeutics that target anabolic pathways involved in skeletogenesis is of great importance with regard to disease resulting in bone loss, or in cases of impaired bone repair. This review aims to summarize recent developments in this area.

**Recent Findings:**

A greater understanding of how drugs that modulate signaling pathways involved in skeletogenesis exert their efficacy, and the molecular mechanisms resulting in bone formation has led to novel pharmacological bone repair strategies. Furthermore, crosstalk between pathways and molecules has suggested signaling synergies that may be exploited for enhanced tissue formation.

**Summary:**

The sequential pharmacological stimulation of the molecular cascades resulting in tissue repair is a promising strategy for the treatment of bone fractures. It is proposed that a therapeutic strategy which mimics the natural cascade of events observed during fracture repair may be achieved through temporal targeting of tissue repair pathways.

## Introduction

Bone tissue has a remarkable capacity for scar-free repair following fracture. This is due to a complex interplay of signaling pathways that recapitulate many aspects of embryonic skeletal development [1, 2], which results in the coordinated regeneration of bone defects. Nevertheless, in up to approximately 10% of all cases, a bone fracture will experience delayed repair with the potential to progress to non-union, with certain bones having a higher risk of non-union, such as the tibia (up to 18.5%) [3]. A non-union is defined as the permanent failure of bone healing after 9 months with no progressive signs of repair in three consecutive months [4]. Risk factors for delayed or non-union can include characteristics of the injury, such as tissue loss or open fracture and surgical issues, including poor stabilization or infection. Host factors, however, carry a significant risk for the development of non-union and include smoking, metabolic disorders, and medication that may influence tissue repair [5]. It is therefore clear that each case of non-union may have its own unique cause, or combination of causes, and as such it is important to assess underlying mediators and stratify patients based on this. Nevertheless, a common requirement for the successful repair of a non-union fracture is the stimulation of the body’s intrinsic mechanisms for tissue repair. Currently, this is achieved through bone grafting, with autologous sources of tissue representing the gold standard, and is successful in approximately 50–80% of cases [6]. Although this technique is the standard of care, it is associated with many limitations and complications, including tissue availability, donor site pain, morbidity and infection [7]. The development of osteoanabolic drugs for the treatment of osteoporosis has created an alternative strategy for the augmentation of fracture repair, while antiresorptive agents such as bisphosphonates and denosumab appear to have some efficacy in promoting aspects of fracture repair (reviewed in [8]). Additionally, bone morphogenetic protein (BMP)-containing devices have been shown to stimulate bone formation and mediate spinal fusion [9] and non-union repair [10]; however, their use in high concentrations has been associated with an increased cancer risk [11], although this is currently disputed [12]. This review aims to summarize the most recent breakthroughs in anabolic strategies for fracture repair with a focus on preclinical data relating to key evidence that modulation of pathways involved in skeletogenesis can improve and indeed rescue fracture repair processes.

## Fracture Repair

**Fig. 1:**
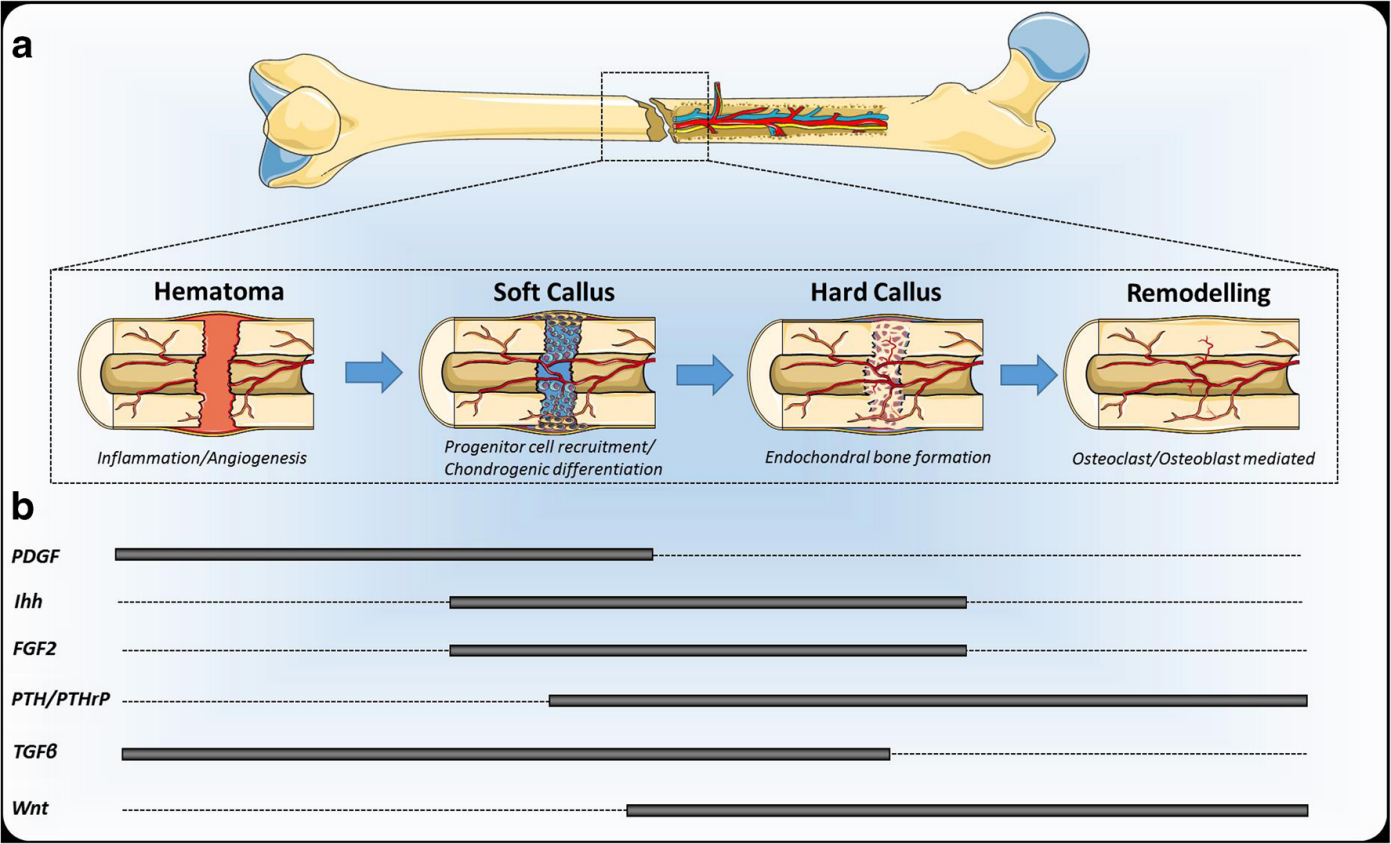
Main stages of long bone fracture repair and associated anabolic signaling pathways. **a** Long bone fractures generally heal through a process of endochondral ossification which progresses through a cartilaginous template. Stem cells are recruited from the periosteum and differentiate toward a hypertrophic chondrocyte phenotype; the matrix surrounding these cells subsequently serves as a scaffold for new bone formation. The process is completed through remodeling events controlled by osteoclasts and osteoblasts resulting in scar-free healing (created and adapted from Servier Medical Art). **b** Major anabolic signaling pathways which have recently been the focus of pharmaceutical targeting indicating their temporal contribution to the tissue formation processes. PDGF stimulates angiogenesis, macrophage recruitment/activation, and mesenchymal progenitor expansion [99]. Ihh plays a role in progenitor recruitment [90••], chondrocyte proliferation and PTHrP expression during endochondral ossification [100, 101]. FGF2 is a potent mitogen for mesenchymal progenitors as well as osteoprogenitors and chondroprogenitors [102, 103]. PTH can exert its function on several stages of fracture repair including cartilage formation, endochondral ossification, and remodeling [104], while PTHrP is known to control chondrocyte hypertrophy [100]. TGFβ signaling is involved in many stages of fracture repair, including the stimulation and proliferation of immune and mesenchymal cells, matrix synthesis, angiogenesis, and regulation of resorption (reviewed in [105]). Enhanced canonical Wnt signaling stimulates osteoprogenitor proliferation, chondrocyte hypertrophy, and decreases bone remodeling [104]

Most fractures repair through a process of endochondral ossification, in a near identical series of morphological steps to embryonic long bone development. The main exception to this is the initial production of a fracture hematoma and the presence of an inflammatory environment [1]. The hematoma is progressively replaced by a cartilaginous callus through condensation of mesenchymal cells from the periosteum in a process controlled by the concerted actions of numerous growth factors, including transforming growth factor beta 1 (TGFβ1), BMPs, fibroblast growth factors (FGFs), stromal-derived factor 1 alpha (SDF1α), and platelet-derived growth factor (PDGF) [13–17]. The importance of the periosteum in the fracture repair cascade has been extensively reviewed elsewhere [18]. The chondrocytes within the fracture callus terminally differentiate to hypertrophy, producing a mineralized matrix that acts as a scaffold for bone formation. This stage and subsequent osteoblast differentiation for bone formation is controlled, in part, through the Wnt/β-catenin pathway [19–21]. Following the progressive replacement of the mineralized cartilage matrix with bone tissue, a series of remodeling events controlled by osteoclasts and osteoblasts re-establish bone contiguity without the formation of a scar. The main exception to this process occurs if the fracture is mechanically stabilized through fixation; in this instance, there is no cartilage formation and the fracture heals through the action of osteoclasts cutting cones across the fracture line and direct (intramembranous) bone formation through the action of osteoblasts [22]. Fig. 1 details the process of long bone fracture repair and the major anabolic signaling pathways associated with each stage.

## Augmenting Fracture Repair with Parathyroid Hormone

Parathyroid hormone (PTH) is an 84 amino acid hormone produced in response to hypocalcemia, and its primary role is to increase serum calcium levels to restore ion balance. This is achieved via the indirect (osteoblast RANK/OPG) stimulation of osteoclastogenesis which in turn sequesters calcium from the skeleton [23]. In addition to this, intermittent exposure to recombinant PTH can promote bone mass accrual [24] and this approach has since has been refined to a 34 amino acid active peptide (teriparatide; PTH(1–34)), which elicits the same effects as full length PTH on the skeleton [25]. This peptide has been shown to stimulate an osteoanabolic response upon intermittent administration in both preclinical models and humans [26, 27]. Due to this activity, teriparatide was approved by the Food and Drug Administration (FDA) as the first bone anabolic agent in November 2002 for the treatment of male hypogonadal or idiopathic osteoporosis and osteoporosis in postmenopausal women at high risk of fracture. In addition to its application in osteoporosis, there is a growing body of evidence that indicates potential efficacy in the treatment of non-union bone fracture. Of interest, intermittent PTH(1–34) has been shown to promote fracture repair and improve union rate (by 59% compared to placebo) in osteopenic rats [28]. Additionally, the effect of intermittent PTH(1–34) has been investigated in the context of fracture repair in non-human primates where a dose-dependent decrease in callus porosity was observed following treatment. It was concluded that this may help to restore mechanical properties of the fracture and therefore accelerate bone healing [29].

A number of studies have been published relating to the use of teriparatide clinically for the augmentation of fracture healing. For example, 20 μg doses of teripartatide administered to female patients with a fracture of the distal radius reduced the mean time to radiographic healing to 7.4 weeks, compared with 9.1 weeks in the placebo group while doses of 40 μg had no effect, thus disproving the primary hypothesis that “teriparatide 40 μg would shorten the time to cortical bridging” [30]. Interestingly, the authors state that the apparent efficacy with 20 μg doses “should be interpreted with caution and warrants further study.” Another group reported the administration of PTH(1–84) (100 μg/day) to postmenopausal women that had suffered a pelvic fracture resulted in complete bridging of the fracture after 7.8 weeks, compared to 12.6 weeks in the control group. Encouragingly, all of the patients in the treatment group had healed after 8 weeks, compared to fewer than 10% in the control group [31]. Positive results have also been reported in studies and case reports using teriparatide in delayed and non-union fractures. Indeed, Bukata and colleagues reported 93% success in achieving union in a cohort of 145 patients with a high incidence of fracture complications (88% had failed a revision surgery, had a non-union, were elderly, or had comorbidities known to affect fracture repair), following 12 weeks of treatment with 20 μg teriparatide [32, 33]. It was noted however that repair of sites consisting of trabecular-rich regions was more rapid than those that were predominantly cortical in nature. Further positive case reports have also been published on the use of teriparatide to achieve repair in cases of delayed/non-union, or in cases where risk factors for delayed union are present (e.g., smoking) [34–39]. It should be noted that at this time, neither PTH nor any of its derivatives have been approved for the treatment of bone fracture and all the above studies represent off-label use.

Despite positive clinical evidence supporting the use of PTH for fracture repair, the precise mechanism by which it achieves its osteoanabolic response is less clear. Indeed, recent literature has indicated novel biology associated with administration of intermittent PTH. Through conditional knockout of PTH/PTH-related peptide (PTHrP) in the limbs of developing mice, Fan and colleagues [40] reported a shift from bone formation towards high marrow adiposity and bone resorption, which the authors propose is due to increased RANKL secretion from marrow adipocytes. Interestingly, intermittent PTH administration reduces marrow adiposity, suggesting that PTH has the ability to control mesenchymal cell fate [40]. Another interesting concept when considering the effect of intermittent PTH on progenitor populations to promote bone formation/repair was uncovered through work investigating quiescent bone lining cells [41]. Lineage tracing experiments utilizing Dmp1CreERt2Rosa26R mice as a reporter allowed the monitoring of mature osteoblasts and their descendants by pulse chase. Through this technique, the authors could monitor the conversion of flat quiescent bone lining cells to cuboidal cells expressing collagen type 1 and osteocalcin, two of the major components of the organic bone matrix. This study therefore indicated that intermittent PTH treatment has the ability to increase osteoblast number by converting lining cells to mature osteoblasts in vivo [41].

In the context of cortical bone formation, which is critical for long bone repair, the effect of PTH on periosteal progenitor cells is of key importance. Indeed, periosteal progenitor cells are responsible for the formation of the fracture callus through chondrogenic differentiation, and also donate osteoblasts for later stages of the repair process. It has recently been shown that PTH has the ability to enhance the proliferation and differentiation of periosteal progenitors in murine models, potentially through Wnt/β-catenin signaling, with the net result of accelerating fracture repair [42]. This was due to enhanced bone formation, without discernible effects on the cartilage phase of fracture repair. This response was however reduced in aged mice, where incomplete resolution of the fracture callus was apparent at 42 days post-fracture [42]. Interestingly, intermittent administration of PTH(1–34) has been shown to have differing effects in trabecular and cortical bone. In trabecular bone, the treatment elicited an increase in osteoblast number; however, there was no effect on trabecular osteocyte sclerostin expression [43]. Conversely, in cortical bone, intermittent administration of PTH(1–34) significantly reduced the number of sclerostin-positive osteocytes; however, this treatment had no effect on endosteal osteoblast number. This had the net effect of increasing trabecular bone parameters, with no effect on the cortical compartment. The finding is in line with data suggesting that the anabolic effect of PTH is not dependent on sclerostin downregulation and is linked to other mechanisms [44••]. Interestingly, this finding may provide some insight into the potential bias toward fracture repair in trabecular sites reported in the Bukata et al. clinical study [33].

Abaloparatide, which is an analog of human PTHrP(1–34), has recently gained FDA approval for the treatment of postmenopausal osteoporosis [45]. This new drug appears to have a similar mechanism of action (MOA) to teriparatide, however, it induces faster increases in bone mineral density (BMD) with larger gains at 6 months, and additional gains at sites such as the hip when compared to teriparatide [46••]. Preclinical studies in mice indicate that PTHrP has the ability to accelerate fracture repair though enhancement of callus formation and promotion of cell differentiation [47, 48]. The authors await data from the field regarding the efficacy of abaloparatide and PTHrP analogues on fracture repair in other models, and more importantly the data for clinical efficacy.

## Modulation of Fracture Repair Through Wnt Signaling

The Wnt pathway is an evolutionary conserved signaling system that controls cell behavior and tissue formation, and has a key role in skeletogenesis. Although there are three Wnt signaling pathways (canonical, non-canonical planar cell polarity, and Wnt-calcium), in the context of bone targeting therapeutics, this review will concentrate on the canonical, or β-catenin dependent pathway. The canonical Wnt pathway is activated following binding of a Wnt protein to a Frizzled receptor and the LRP5/6 Wnt co-receptors, thus promoting stabilization and nuclear translocation of β-catenin to activate Wnt target gene expression. The importance of this pathway in bone homeostasis is evidenced through the identification of loss and gain of function mutations in LRP5 that resulted in low (osteoporosis pseudoglioma syndrome) [49] or high bone mass [50], respectively. Additionally, high bone mass diseases have been identified and linked to the loss of the protein sclerostin, a natural inhibitor of the Wnt signaling pathway, which binds the LRP5/6 receptors. Two similar rare diseases, which share highly similar high bone mass phenotypes, have been identified and characterized—namely sclerosteosis [51, 52] and Van Buchem disease [53, 54]. These observations led to the formulation of neutralizing antibodies against sclerostin, which deliver robust increases in BMD through a dual action in promoting osteoblast differentiation while suppressing osteoclast formation [55••, 56, 57]. Interestingly, another Wnt antagonist, Dkk1, which is temporally expressed during fracture repair and upregulated in cell populations derived from human non-union fibrous tissues [58], is also upregulated in response to treatment with sclerostin antibody [59].

Sclerostin neutralization has been previously shown to accelerate fracture repair in preclinical models, including non-human primates and rats [60, 61], with similar data associated with the inhibition of Dkk1 in rodents [62]. In an attempt to maximize the bone-forming response for fracture repair, a bi-specific antibody targeting both antagonists was formulated, and delivered significantly higher serum biomarker levels associated with bone formation in non-human primates and enhanced rat fracture repair to a greater extent than sclerostin or Dkk1 antibody alone [63••]. Indeed, administration of this bi-specific antibody dose-dependently increased callus bone volume, cross-sectional area, and torsional strength compared to sclerostin antibody (Scl-Ab) alone, which even at a high dose of 75 mg/kg resulted in non-significant increases in these parameters equivalent to 25-fold lower doses of the bi-specific antibody. It was concluded that this was not a dose-dependent effect and instead a synergy due to the combined neutralization of the two Wnt antagonists. It is hypothesized that the synergy observed is due, at least in part, to the skeleton producing Dkk1 in response to sclerostin neutralization in an attempt to self-regulate the increase in bone formation, which when neutralized with this bi-specific antibody produces an effect that is greater than inhibition of either antagonist alone. Interestingly, this synergy between Dkk1 and sclerostin has also been reported through the use of transgenic mice where bone-selective deletion of both proteins increased BMD to a greater extent than the additive effect of each alone, although the effect on fracture repair was not reported [64]. Recently, another Wnt modulator, SOSTDC1, which also functions as a BMP antagonist, has been implicated in the fracture repair process in mice, whereby loss of function promotes fracture callus formation and bone repair [65]. It is, however, unclear how this compares to sclerostin or/and Dkk1 inhibition or indeed whether antibody-targeted neutralization would be comparable to genetic deletion. In summary, although published data show that increased canonical Wnt signaling by neutralizing one or more Wnt inhibitors enhances fracture healing in union and non-union animal models, its benefits in a clinical setting is currently unknown.

## Modulating the Transforming Growth Factor Beta Superfamily to Promote Fracture Repair

The transforming growth factor beta (TGFβ) superfamily of secreted factors consists of over 30 members including Activins, Nodals, bone morphogenetic proteins (BMPs), and growth and differentiation factors (GDFs). These factors have key roles from early development through to adult tissue homeostasis, with dysfunctional activity being attributed to pathology. TGFβ superfamily members signal through cell-surface serine/threonine kinase receptors and influence the phosphorylation status of Smad proteins with the result of promoting specific gene expression. In the context of bone development and repair, it is known that members of this family are involved in chondrogenesis, osteoblastogenesis, and osteoclastogenesis. As with Wnt signaling, numerous antagonists modulate this signaling pathway; however, unlike the Wnt pathway, most efforts in skeletal repair have been made in promoting signaling through addition of exogenous ligands. Indeed, much preclinical and clinical research has centered on BMP2 and BMP7, which has been reviewed extensively elsewhere and as such, will not be discussed in any detail [66]. It is however important to state that conflicting data has been reported relating to their efficacy in promoting repair of non-union fractures and safety concerns have limited their recent use. As such, efforts have centered on limiting off-target effects associated with supplying supraphysiological doses of exogenous BMP ligands to the fracture site. One such mechanism to reduce drug load would be through simultaneous or sequential stimulation of multiple pathways involved in skeletogenesis, thus better replicating the natural cascade of events observed during fracture repair. In line with this, literature suggests that simultaneously promoting BMP signaling and Wnt signaling appears to be more effective than the administration of BMP alone. Tinsley and colleagues reported impressive healing of critical sized rat femoral defects with systemically administered Scl-Ab and locally implanted BMP2 when compared to BMP2-implanted animals. Indeed, following 12 weeks of treatment, all animals receiving Scl-Ab and BMP had 90% greater bone volume and improved biomechanical properties compared to the BMP group alone [67].

Advances have also been made in relation to the delivery of BMPs to the site of fracture, mainly through the use of carriers or through the use of gene transfer. Recently, it has been shown that ultrasound-mediated BMP6 transfer and expression is able to promote bone healing in a minipig critical sized defect model. In this study, endogenous mesenchymal cells were recruited into a collagen matrix followed by ultrasound-mediated gene transfer, which led to complete radiographic and functional healing in all animals after 6 weeks [68]. In a further effort to limit off-target effects of BMPs, ex vivo priming of periosteal cell populations involved in the fracture repair process with BMP2 prior to implantation in a critical size murine tibia defect has been reported. This methodology resulted in the endogenous production of physiological levels of growth and differentiation factors and robust tissue formation in a process that mimicked the fracture repair cascade [69].

In contrast to supplying soluble ligands, TGFβ receptor fusion proteins have been investigated for their effect on skeletogenesis mainly due to the role that certain ligands have in promoting bone loss. For example, it has been reported that Activin A can suppress osteoblast mineralization while promoting osteoclast formation [70, 71]. As such, ligand sinks (receptor extracellular domain-Fc) have been formulated from the major receptors of the TGFβ superfamily for the potential treatment of bone disease. Fc fusion proteins of type I BMP receptors (BMPRIA and BMPRIB) [72] and type II receptors (ActRIIA, ActRIIB) have been shown to stimulate bone mass accrual. Indeed, a soluble ActRIIA molecule was able to promote bone formation during fracture repair; however, the bone was of lesser quality compared to controls, which may be due to the negative effect of this molecule on osteoclast formation [73]. Similarly, ActRIIB-Fc has been shown to promote bone formation in mice [74]; however, its specific role in augmenting the fracture repair process has yet to be investigated. Nevertheless, it has been shown to increase bone mass in a murine model of Duchene muscular dystrophy, a disease which is characterized by muscle degeneration and a high incidence of fracture [75].

## Other Pathways with Potential Applications to Non-union Fracture Repair

### Platelet-Derived Growth Factor

Platelet-derived growth factor (PDGF) is a potent pro-angiogenic, mitogenic, and chemotactic factor produced by multiple cell types including vascular smooth muscle cells, macrophages, and macrophage descendants, and is known to play a number of roles within the skeletal system [76]. PDGF exists as a dimeric protein with five specific forms; AA, BB, AB, CC, and DD. The A and B forms of the protein are secreted as active ligands, whereas the C and D forms are secreted as latent forms and subsequently undergo activation by extracellular proteolytic action [77]. Recombinant human (rh) PDGF-BB is FDA approved for the treatment of chronic foot ulcers in diabetic patients, regeneration of infection-associated loss of alveolar bone, and foot and ankle fusion [78], which will be discussed in more detail later.

PDGF-BB appears to have a major function within the skeletal system, including the regulation of the initiation of fracture repair. This role is through its concerted action on angiogenesis and mesenchymal cell recruitment, proliferation, and differentiation [76], which appear in part to be due to its effect on the JNK and ERK pathways [79]. PDGF-BB also plays a key role in bone development where its inhibition results in reduced BMD at both trabecular and cortical sites [80]. Numerous early preclinical studies have shown efficacy of PDGF to augment fracture repair, which have been reviewed elsewhere [81]. More recently, it has been reported that the transplantation of hematopoietic progenitors overexpressing PDGF-BB into mice increased trabecular bone formation and trabecular connectivity, and decreased cortical porosity, resulting in a 45% increase in bone strength [82]. This effect did, however, appear to be dose-dependent as the use of stronger gene promoters resulted in osteomalacia. It was concluded that the increase in bone parameters was due to the anabolic action of PDGF-BB on mesenchymal stem cells in the bone marrow microenvironment.

RhPDGF has been used clinically for hindfoot fusion in patients at high risk of non-union [83]. Indeed, a recent clinical trial investigating ankle and hindfoot fusion using 0.3 mg/mL rhPDGF-BB/β-TCP-collagen compared to autologous bone grafting reported that 84% of the rhPDGF-BB/β-TCP-collagen-treated patients achieved complete fusion compared to 65% of the autograft-treated patients. Furthermore, a higher percentage (91 vs. 78%) also achieved clinical success with a quicker fusion time (14.3 ± 8.9 vs. 19.7 ± 11.5 weeks) when comparing rhPDGF-BB/β-TCP-collagen patients vs. autograft patients [84]. Despite this success, no reports of the use of rhPDGF-BB to treat non-union fractures of long bones have been published to date.

### Indian Hedgehog

The Hedgehog (Hh) proteins are evolutionary conserved across species and include Sonic Hh (Shh), Desert Hh (Dhh), and Indian Hh (Ihh), all of which signal through their receptors Patched and Smoothened. Hh proteins have a fundamental role in skeletal development in the context of embryonic limb formation and postnatal bone growth. For example, Shh is expressed in the limb bud and specifies positional values for digit formation as well as the width of the limb bud itself (reviewed in [85]). In the context of postnatal longitudinal bone growth, Ihh is expressed in the prehypertophic zone of the growth plate and forms a negative feedback loop with PTHrP to regulate the pace of chondrocyte differentiation and therefore the rate of bone growth; interestingly, Ihh has also been reported to directly affect hypertrophy in the absence of PTHrP [86]. Ihh also appears to be involved with intramembranous ossification, with knockout mice displaying reduced cranial size [87]. In the context of this review, both Ihh and Patched are upregulated at early stages of fracture repair indicating their role in later tissue-forming events [88]. Interestingly, in a rabbit tibial defect model, the implantation of a complex of MSCs engineered to overexpress Ihh in a hydroxyapatite scaffold promoted bone repair more effectively than MSCs/scaffold alone [89]. In agreement, Ihh has recently been shown to be downregulated in dysfunctional skeletal stem cell niches in diabetic mice [90••]. The study reported that suppression of Hh signaling during fracture repair suppressed the expansion of skeletal stem cells, which retarded the normal fracture repair process. Interestingly, diabetes is a major risk factor for impaired fracture repair resulting in delayed and non-union clinically (reviewed in [91]). The investigators reported that the precise delivery of Ihh to the fracture site in a slow release hydrogel restored the repair process. It remains to be proven whether Ihh deficiency is a major cause of non-union in non-diabetic individuals and if so whether this may be a therapeutic option to treat all non-union fractures.

### Fibroblast Growth Factor 2

Fibroblast growth factor 2 (FGF2) or basic FGF is one of a large family of growth factors that plays a role in angiogenesis and mitogenesis of multiple cell types. FGF2 signals through two (FGFR2 and FGFR3) of the four known FGF receptors (FGFRs). This signaling pathway is intrinsically linked with skeletogenesis as activating mutations in FGFR3 cause achondroplasia, hypochondroplasia, or thanatophoric dysplasia, manifesting in short stature [92]. Conversely, inactivating mutations in FGFR3 cause tall stature [93]. Although this phenotype is due to the effect of these mutations on the growth plate, FGF2 is known to be produced by osteoblasts and stored in the bone matrix. Additionally, FGF2 is expressed during the early stages of fracture repair and the receptors are expressed throughout the fracture callus [94]. Contradictory reports on the efficacy of FGF2 to promote bone repair in early preclinical models exist [95]; however, they will not be discussed in detail here. Recently, a low molecular weight (LMW) isoform of FGF2 has been investigated for its role in fracture repair. Interestingly, targeted overexpression of this isoform in the osteoblast lineage of mice caused an increase in BMD, whereas the opposite was true for all other isoforms [96]. Furthermore, BMP2 could synergize with LMW FGF2 to heal a critical sized cranial defect in LMW FGF2 transgenic mice [97]. The authors also stated that the enhanced calvarial healing was due to increased canonical Wnt signaling. In a later publication, the authors also tested the LMW FGF2 transgenic mice for tibial healing and concluded that LMW FGF2 also accelerated the fracture healing process of long bone defects [98]. Unfortunately, BMP was not co-implanted in this study so it is not evident whether similar synergies occur as seen in the calvaria. They did, however, report an increase in PDGF-BB which may contribute to the enhanced repair process. It remains to be seen whether this LMW FGF2 will deliver robust fracture repair if delivered exogenously in other animal models.

## Future Perspectives

As discussed, there is a wealth of preclinical research and clinical trials ongoing to deduce the efficacy of osteo/chondro anabolic agents on the fracture repair process. The aim of this effort is to discover an agent that has the ability to overcome the failure of the biological tissue-forming cascades observed in fracture non-union. Previous preclinical studies have shown that synergies exist between signaling pathways such as BMP/Wnt, BMP/FGF2, and FGF2/PTH, that when stimulated drive more efficient fracture repair than either factor alone. It is our belief that targeting multiple anabolic signaling pathways, either simultaneously or sequentially, will induce more efficient expansion of skeletal stem cells from their niches and result in coordinated tissue formation. Through the careful selection of these pathways, a therapeutic strategy that mimics the natural cascade of events observed during fracture repair may be achieved. It is envisaged that advances in disruptive technologies that allow intracellular targeting of factors important in de novo skeletal tissue formation, along with novel antibody discovery methodologies allowing the creation bi-specific reagents, will uncover therapeutic strategies that can initiate the body’s natural repair processes.
